# More evening preference is positively associated with systemic inflammation in prediabetes and type 2 diabetes patients

**DOI:** 10.1038/s41598-018-34045-y

**Published:** 2018-10-26

**Authors:** Hataikarn Nimitphong, Apichana Mahattanapreut, La-or Chailurkit, Sunee Saetung, Nantaporn Siwasaranond, Rungtip Sumritsopak, Thunyarat Anothaisintawee, Ammarin Thakkinstian, Lara R. Dugas, Brian T. Layden, Sirimon Reutrakul

**Affiliations:** 1 Division of Endocrinology and Metabolism, Mahidol University, 270 Rama VI Rd, Ratchathewi, Bangkok, 10400 Thailand; 2 Department of Family Medicine, Faculty of Medicine Ramathibodi Hospital, Mahidol University, 270 Rama VI Rd, Ratchathewi, Bangkok, 10400 Thailand; 3 Section for Clinical Epidemiology and Biostatistics, Faculty of Medicine Ramathibodi Hospital, Mahidol University, Bangkok, Thailand; 4 Public Health Sciences, Loyola University Chicago Stritch School of Medicine, Maywood, IL USA; 5 Division of Endocrinology, Diabetes and Metabolism, Department of Medicine, University of Illinois at Chicago, Chicago, IL USA; 6 Jesse Brown Veterans Affair Medical Center, Chicago, IL USA

## Abstract

Currently it is not known whether morningness-eveningness preference in non-night shift working population is associated with systemic inflammation. This study investigated the relationship between morningness-eveningness and systemic inflammation, as measured by high-sensitivity C-reactive protein (hs-CRP) in 163 non-night shift working patients with abnormal glucose tolerance (86 type 2 diabetes and 77 prediabetes). Morningness-eveningness was assessed by Composite Scale of Morningness, and participants were screened for Obstructive sleep apnea (OSA). Sleep duration, efficiency, and variability were obtained using actigraphy, and depressive symptoms and dietary patterns were also captured. Participants’ mean age was 54.7 ± 10.4 years and median hs-CRP was 1.39 (interquartile range 0.82, 3.33) mg/L. More evening preference was significantly associated with higher natural log transformed (ln) hs-CRP (B = −0.051, p = 0.001). Diabetes status, glycemic control, OSA severity, sleep duration, caloric consumption and timing were not related to hs-CRP. After adjusting for age, sex, body mass index, depressive symptoms, sleep efficiency, sleep variability, percentage of daily caloric intake from protein, and statin use, more evening preference was independently associated with higher ln hs-CRP (B = −0.032, p = 0.014). In summary, in non-night shift working patients with abnormal glucose tolerance, more evening preference was independently associated with higher systemic inflammation. This finding underscore the importance of circadian regulation on cardiovascular health.

## Introduction

The circadian system is known to play a crucial role in hormonal regulation and energy metabolism^1^. Circadian misalignment occurs when the endogenous circadian rhythms are not synchronized with either the environment or each other. This can happen when behaviors such as wake time, sleep time, and meals are not occurring at an appropriate time relative to the timing of the central circadian clock, located in the hypothalamus, and/or relative the external environment, particularly the light-dark cycle^2^. For example, during experimental circadian misalignment, when healthy volunteers alter their habitual eating and sleep times, these changes result in impaired glucose metabolism, elevated blood pressure and elevated levels of high-sensitivity C-reactive protein (hs-CRP), reflecting increased systemic inflammation which could represent cardiovascular risks if persists in a long term^3–5^. Indeed night shift workers, who typically experience circadian misalignment as they eat and are active during biological night and sleep during their biological day, are found to have increased risk of adverse health outcomes including diabetes^6^, hypertension, obesity^7^ and cardiovascular disease^8,9^. It is possible that the increased systemic inflammation seen in shift workers may be responsible in their increased risk of adverse health outcomes^10^.

In non-night shift workers, individuals with more evening preference or “later chronotype”, generally present with preferred later sleeping and meal timing, typically experience a mild form of circadian misalignment^11,12^. The time of day during which individuals prefer to sleep or perform daily activities denotes morningness– eveningness or chronotype. In those with more evening preference or later chronotype, their timing of activities as imposed by the society may not coincide with the timing of their endogenous biological clock, which could lead to chronodistruption. Recent evidence suggested that chronodisruption could be detrimental to metabolic health as evening types have been described to be associated with cardiometabolic disorders, including metabolic syndrome^13^, diabetes^14^, hypertension^14^ and cardiovascular disease^11^. In the recent analysis of the UK Biobank cohort, evening types had a 10% increased risk of all-cause mortality after a 6.5-year follow up compared to morning types^11^. In addition, among those with type 2 diabetes, later chronoytpe was observed to be associated with poorer glycemic control^15,16^. Matching social schedules to the person’s chronotype could have an important metabolic impact as it was found that later chronotypes had the lower diabetes risk if their schedules included night shifts, compared to if they only worked daytime schedules^17^.

Despite these data, no studies have explored whether more evening preference is associated with increased systemic inflammation in non-night shift workers, especially in people with abnormal glucose tolerance who are at high risk of developing cardiovascular events. If proven, this could be one of the mechanisms linking more evening preference to adverse cardiovascular outcomes. High-sensitivity CRP is an inflammatory marker that predicts cardiovascular disease and mortality in those with and without diabetes^18,19^. CRP is predominantly synthesized in the liver in response to proinflammatory cytokines (i.e. interleukin (IL)-1, IL-6, IL-17 and tumor necrosis factor-α), and is a marker for infection, increased in response to injury as well as systemic inflammation^20,21^. Moreover, hs-CRP has a long half-life, is stable and its assay yields reproducible results^20,22^. Therefore, the aim of this study was to explore the independent relationship between morningness-eveningness preference and hs-CRP in patients with diabetes and prediabetes, while comprehensively examining covariates that could affect circadian regulation and inflammatory process including sleep characteristics and meal timing, obstructive sleep apnea (OSA) and depressive symptoms^23,24^. We hypothesized that more evening preference, as assessed by the Composite Scale of Morningness (CSM), was independently associated with higher systemic inflammation in this patient group.

## Results

A total of 163 patients (86 with type 2 diabetes and 77 with prediabetes) were included (see Table 1). Mean (SD) age was 54.7 (10.4) years, and 67.5% of the participants were female. A majority of the participants had hypertension, dyslipidemia and were using statins. The median (IQR) hs-CRP was 1.39 (0.82, 3.33) mg/L, while the mean CSM score was 44.2 (5.4), indicating more morningness preference of this population. Obstructive sleep apnea (OSA) was found in 76.1% of the participants with a median apnea hypopnea index (AHI) of 10.5 (5.2, 18.8). Sleep parameters obtained from approximately 7-day actigraphy recordings including sleep duration, sleep efficiency, sleep timing and sleep variability (standard deviation of midsleep time), along with dietary parameters (daily caloric intake, their distribution and meal timing) are also shown (Table 1).

**Table 1:** Participants’ demographics, morningness-eveningness, sleep and dietary variables, and hs-CRP.

	Results
**Demographic, glycemic and hs-CRP**	
Age (years)	54.7 (10.4)
Female (n) (%)	100 (67.5%)
BMI (kg/m^2^)	27.7 (4.6)
Diabetes (vs. prediabetes) (n) (%)	86 (52.8%)
HbA1c (%)	6.79 (1.27)
eGFR (ml/min/1.73 m^2^)*	89.9 (21.9)
Hypertension (n) (%)	104 (63.8%)
Dyslipidemia (n) (%)^†^	138 (84.7%)
Statin use (n) (%)^†^	105 (64.4%)
CESD score^‡^	11.5 (6.4)
hs-CRP(mg/L)	2.50 (2.92) Median 1.39 (0.82, 3.33)
**Morningness-Eveningness preference and sleep characteristics**	
Composite Scale of Morningness score	44.2 (5.4)
Apnea hypopnea index (events/h)	13.9 (12.2), median 10.5 (5.2, 18.8)
Sleep duration by actigraphy (h)^§^	5.99 (0.99)
Sleep efficiency by actigraphy (%)^§^	82.5 (7.9)
Bedtime by actigraphy (hh:mm)	22:49 (1:18)
Sleep end time by actigraphy (hh:mm)	5:48 (1:01)
Midsleep time by actigraphy (hh:mm)	02:26 (0:55)
Standard deviation of midsleep time by actigraphy (h)	0.63 (0.40)
**Dietary characteristics**	
Total daily calorie (kacal/day)	1320 (327)
Breakfast time (hh:mm)^†^	7:44 (1:16)
Lunch time (hh:mm)^||^	12:32 (0:41)
Dinner time (hh:mm)^||^	18:29 (0:52)
Percent daily caloric intake at breakfast	28.8 (6.7)
Percent daily caloric intake at lunch	31.5 (7.3)
Percent daily caloric intake at dinner	30.8 (7.3)
Percent daily caloric intake from fat	30.5 (6.7)
Percent daily caloric intake from carbohydrate	55.5 (7.3)
Percent daily caloric intake from protein	15.9 (2.4)

^*^n = 156, ^†^n = 161, ^‡^n = 155, ^§^n = 162, ^||^n = 160.

Data are expressed as mean (SD) or frequency (%).

Table 2 showed the associations between participants’ characteristics and ln hs-CRP levels. Overall, more evening preference (lower CSM score) was significantly associated with higher hs-CRP (B = −0.051, p = 0.001). In addition, younger age (p < 0.001), being female (p = 0.008), higher BMI (p < 0.001), higher sleep variability (p = 0.023), and higher percentage daily caloric intake from protein (p = 0.049) were also significantly associated with higher hs-CRP. While not significant, statin use (p = 0.064), greater depressive symptoms (CESD score, p = 0.054), and lower sleep efficiency (p = 0.064) showed a strong trend with hs-CRP. Diabetes status, hypertension, dyslipidemia, HbA1c, sleep duration and timing, meal timing, daily caloric intake and their distribution were not related to hs-CRP.

**Table 2:** Simple linear regression analysis between demographics, morningness-eveningness, sleep and dietary variables, and ln hs-CRP (mg/L).

	Association with hs-CRP	
	B	p
**Demographic and glycemic characteristics**		
Age (years)	−0.028	<0.001
Female	0.452	0.008
BMI (kg/m^2^)	0.113	<0.001
Diabetes (vs. prediabetes) (n) (%)	−0.050	0.757
HbA1c (%)	0.055	0.385
eGFR (ml/min/1.73 m^2^)*	0.006	0.123
Hypertension	−0.059	0.726
Dyslipidemia^†^	−0.106	0.651
Statin use^†^	−0.315	0.064
CESD score^‡^	0.025	0.054
**Morningness-Eveningness preference and sleep characteristics**		
Composite Scale of Morningness score	−0.051	0.001
Apnea hypopnea index (events/h)	0.112	0.101
Sleep duration by actigraphy (h)^§^	−0.088	0.283
Sleep efficiency by actigraphy (%)^§^	−0.019	0.060
Bedtime by actigraphy (hh:mm)	0.086	0.170
Sleep end time by actigraphy (hh:mm)	0.039	0.625
Midsleep time by actigraphy (hh:mm)	0.092	0.293
Standard deviation of midsleep time by actigraphy (h)	0.459	0.023
**Dietary characteristics**		
Total daily calorie (kcal/day)	0.0001	0.618
Breakfast time (hh:mm)^†^	−0.077	0.235
Lunch time (hh:mm)^||^	−0.072	0.539
Dinner time (hh:mm)^||^	−0.077	0.413
Percent daily caloric intake at breakfast	−0.006	0.633
Percent daily caloric intake at lunch	0.013	0.243
Percent daily caloric intake at dinner	0.001	0.906
Percent daily caloric intake from fat	0.005	0.682
Percent daily caloric intake from carbohydrate	−0.007	0.521
Percent daily caloric intake form protein	0.065	0.049

*n = 156, ^†^n = 161, ^‡^n = 155, ^§^n = 162, ^||^n = 160.

B = unstandardized coefficient.

After adjusting for age, sex, BMI, statin use, CESD, sleep efficiency, sleep variability and percentage of daily caloric intake from protein, more evening preference was independently associated with higher hs-CRP (B = −0.032, p = 0.014) (Table 3). In addition, a higher BMI (B = 0.109, p < 0.001) and being female (B = 0.377, p = 0.010) were significantly associated with higher hs-CRP while statin use was significantly associated with lower hs-CRP (B = −0.421, p = 0.004). The adjusted R-squared of the model was 0.326.

**Table 3:** Multivariate analysis by backward elimination with ln hs-CRP as an outcome.

Variables	Coefficient	SE	t	P value	95% CI
Female	0.377	0.143	2.63	0.010	0.093, 0.661
BMI	0.108	0.015	7.18	<0.001	0.079, 0.139
Statin	−0.421	0.143	−2.95	0.004	−0.703, −0.139
Composite Scale of Morningness Score	−0.032	0.013	−2.50	0.014	−0.057, −0.007

Adjusted R-squared = 0.326.

In a restricted analysis of either patients with prediabetes or diabetes, we found that in those with prediabetes, more evening preference was significantly associated with higher ln hs-CRP (B = −0.034, p = 0.033) (Table 4). This relationship was attenuated in those with type 2 diabetes (Table 5).

**Table 4:** Multivariate analysis with ln hs-CRP as an outcome in patients with prediabetes.

Variables	Coefficient	SE	t	P value	95% CI
Female	0.558	0.197	2.84	0.006	0.167, 0.951
BMI	0.110	0.021	5.26	<0.001	0.068, 0.152
Statin	−0.285	0.168	−1.70	0.094	−0.619, 0.049
Composite Scale of Morningness Score	−0.034	0.016	−2.18	0.033	−0.065, 0.003

Adjusted R-squared = 0.359.

**Table 5:** Multivariate analysis with ln hs-CRP as an outcome in patients with type 2 diabetes.

Variables	Coefficient	SE	t	P value	95% CI
Female	0.297	0.218	1.36	0.178	−0.137, 0.731
BMI	0.110	0.022	4.98	<0.001	0.066, 0.154
Statin	−0.597	0.286	−2.09	0.040	−1.167, −0.0.27
Composite Scale of Morningness Score	−0.027	0.021	−1.29	0.200	−0.069, 0.015

Adjusted R-squared = 0.295.

## Discussion

In our study, we sought to explore the association between chronotype and hs-CRP levels in non-night shift prediabetes and diabetic patients. Our novel findings indicate that a more evening preference is independently associated with higher ln hs-CRP levels, after considering multiple confounders including sleep characteristics, meal timing and comorbidities. Additionally, this relationship was more pronounced in those with prediabetes only, possibly due to less medication use in this group. These findings are especially relevant given the emerging evidence of detrimental effects of evening preference on cardiometabolic disorder and mortality^11^. Elevated hs-CRP could be one of the underlying mechanisms in this relationship, especially in our patients with abnormal glucose tolerance who are at high risk for cardiovascular events. The estimated effect size of the difference between 10^th^ and 90^th^ percentile of CSM score on hs-CRP was 0.46 mg/L in this cohort. This difference could be clinically significant as the risk for cardiovascular events was incremental among those with hs-CRP <1, 1–3 and >3 mg/L, up to 2.9-fold increase^25^. Another effect size comparison could be done with the use of statin, a medication shown to significantly reduce cardiovascular events, which was associated with an approximately 1 mg/L reduction in hs-CRP^26^. All together, our data underscore the importance of circadian regulation on systemic inflammation in non-night shift workers.

Factors associated with more evening preference may help explain its relationship with hs-CRP. Depending on social obligations, evening types may experience short sleep duration, especially during work days as they have to wake up earlier than desired to conform with social or work schedule. Indeed, insufficient sleep is known to result in increased systemic inflammation^27^. In our cohort, however, sleep duration was not associated with hs-CRP, possibly due to the smaller sample size. Similarly, reduced sleep quality has also previously been found to be more common among evening types^28^, which could, in itself, lead to increased inflammation. We found strong, non-significant trends between sleep efficiency and hs-CRP, but this association was attenuated after adjusting for other covariates. This could also be partly due to a relatively morning preference in our patients. Similarly, depression could additionally be a contributor as evening types were likely to have higher risk of incident depression and greater depressive symptoms than morning types^29–31^. Increasing severity of depression was associated with higher hs-CRP^24^. Our results agree with this finding as depression, as measured by the CESD score, was related to both hs-CRP and CSM score (result not shown).

Sleep variability may also play a role as those with more evening preference often shift their sleep timing between work days, when they have social obligations, and weekends, when they can follow their own rhythm. Increased sleep variability has previously been shown to be associated with increased inflammatory markers including tumor necrosis factor-α (TNF- α)^32^. In our study, variability of midsleep time, the indicator of sleep variability, was significantly correlated with both hs-CRP and more evening preference but was not an independent predictor of hs-CRP. In addition, poor diet quality, a contributor to systemic inflammation, has been described in those with later sleeping time^33,34^, but we could not account for any dietary effects on hs-CRP. Lastly, evening types may experience more light exposure at night due to later sleep timing. Dim light at night was shown to exaggerate inflammation in mice, especially when exposed to a high-fat diet^35^. Our study is limited by lack of light exposure information. It is possible that these behaviors collectively, but not individually, contribute to systemic inflammation in those with more evening preference. It is also possible that the effects of circadian misalignment seen in those with more evening preference are not entirely captured by these variables. In this study, the relationship between more evening preference and higher hs-CRP persisted even after considerations of these factors.

Chronotherapeutic interventions such as bright light therapy and sleep phase advance could possibly offer cardiometabolic benefits in evening type but these studies are currently lacking. Light is known as the primary synchronizer of the circadian system^36^. There is some limited data suggesting positive metabolic outcomes from the use of bright light therapy in overweight or obese population in reducing fat mass and hunger, although these studies did not consider morningness-eveningness as a study factor^37–39^. However, another study found that five hours of morning bright light was detrimental to metabolic health in type 2 diabetes^40^. Thus, whether morning light therapy could reduce systemic inflammation in evening types, especially those with cardiovascular risks, needs to be further explored.

Besides morning light, melatonin supplementation should also be considered with regards to its effect on circadian rhythm. Melatonin is a neurohormone secreted by a pineal gland and the 24 h rhythm of melatonin acts as an internal synchronizer of the circadian system^41,42^. When given in the early evening, a few hours before desired bedtime and earlier than the individual’s endogenous melatonin onset, it can elicit phase advance shifts^43^, and reportedly has anti-oxidant properties^44^. Recently, the effects of 10 mg melatonin, given one hour before bed time for 12 weeks in 60 patients with type 2 diabetes and coronary heart disease were shown to decrease systemic inflammation^45^. Whether melatonin will reduce inflammation associated with more evening preference is currently not known. Simple behavioral modifications may also be promising. In a randomized study of 31 patients with type 2 diabetes with late sleep time (after midnight), three-month sleep education program in a combination with diabetes education led to a significant greater reduction in hs-CRP and interlekin-6 levels, along with decreased plasma glucose, HbA1c, and improved self-reported sleep quality, compared to a conventional diabetes education alone^46^. Lastly, social schedule that matches with individuals’ endogenous timing may alleviate the detrimental effects of evening types. This was shown in the analysis of the Nurses’ Health Study 2, in which evening types had an increased risk of developing diabetes when they worked day shifts, while this risk was attenuated if their work included night shifts^17^. These data support the importance of synchronization between external input to the circadian system (i.e. zeitgeber) and endogenous timing on metabolic health.

The strengths of the current study include comprehensive measurements of morningness-eveningness, hs-CRP, objective sleep measures, and dietary variables exclusively in patients with abnormal glucose tolerance. However, we note several limitations to our study, including the cross-sectional study design which precludes an assumption of a causal relationship. Further intervention studies are needed to establish this possible causal association. In addition, our participants had relatively more morning preference as reflected by their CSM scores. It is known that geographic location, likely due to temperature and sun light exposure, is related to circadian preference with countries closer to the equator being more morning^47,48^. Even though our population had relatively more morning preference, we did see a relationship between CSM and hs-CRP across CSM continuum.

As this study included only Thais, the results may not be applicable to other ethnic groups as cultural differences in diet patterns and other health outcomes may impact these outcomes. Certain variables such as exercise details and light exposure at night were not available for our cohort, a limitation of this study. Similarly, the details of over-the-counter medication use which could affect hs-CRP, such as NSAIDs, were not available. The duration of actigraphy recordings (one week) was also relatively short. Our participants had a high rate of OSA, known to be associated with systemic inflammation and diabetes^49,50^. However, the relationship between more evening preference and higher hs-CRP persisted after considering OSA severity in the current study. Moreover, the reproducibility of our findings in other populations and also with those with less OSA risk needs further investigation. Lastly, other inflammatory markers, such as IL-1 and IL6, will need to be investigated.

In summary, a chronotype reflecting more evening preference in patients with abnormal glucose tolerance who were not night shift workers was independently associated with higher systemic inflammation. These findings support the importance of circadian regulation on cardiovascular health.

## Methods

### Participants

Non-night shift working adults with a clinical diagnosis of type 2 diabetes, and prediabetes who were being followed in the outpatient clinics at the Faculty of Medicine Ramathibodi Hospital, Bangkok were invited to participate. Prediabetes was defined as having fasting plasma glucose between 100–125 mg/dL and hemoglobin A1c < 6.5%^51^. Upon screening, those patients who reported working overnight shifts were excluded. Those patients with cerebrovascular disease, end stage renal disease, congestive heart failure, permanent pacemaker, significant pulmonary disease or cirrhosis, and previous diagnosis of OSA were also excluded. In addition, the use of certain medications (opioids/narcotics, alpha adrenergic blockers, clonidine, methyldopa, nitroglycerin) were also excluded in order to obtain valid results from the OSA diagnostic method utilized (see below). All participants gave written informed consent. The protocol was approved by the Committee on Human Right Related to Research Involving Human Subjects, Faculty of Medicine Ramathibodi Hospital, Mahidol University. The methods were carried out in accordance with the Declaration of Helsinki.

### Demographics, medical history, glycemic control and depressive symptoms

Weight was measured with participants wearing light clothing and no shoes. Height, age, current medications including statin use, history of hypertension and dyslipidemia, estimated glomerular filtration rate (eGFR, within one year), and hemoglobin A1c (HbA1c) levels (within the prior 90 days) were extracted from medical records. HbA1c is a gold standard measurement of glycemic control. Hypertension and dyslipidemia were categorized as “yes” if the participants had a documented diagnoses of these conditions or were taking medications, including statin use, because they have been reported to affect hs-CRP^26,52–55^. Body mass index (BMI) was calculated as weight (kg)/height^2^ (m^2^). Depressive symptoms were assessed by The Center for Epidemiological Studies Depression (CES-D) questionnaire which was validated in Thai language^56,57^.

### Morningness-Eveningness assessment

Morningness-eveningness preference was assessed using the validated Thai version of the Composite Scale of Morningness (CSM)^58,59^. The CSM consists of 13 questions regarding the preferred time individuals would like to wake up and go to bed, preferred time for physical and mental activity, and subjective alertness. The total score ranges from 13 (extreme eveningness) to 55 (extreme morningness).

### Objective sleep measurements

OSA was diagnosed using an FDA-approved portable diagnostic device, WatchPAT 200 (Itamar Medical, Israel), which has been validated against polysomnography in populations with and without diabetes^60,61^. This non-invasive device is shaped similar to a large watch, worn on the non-dominant wrist immediately before bedtime and removed upon awakening in the morning. The device has two probes connecting to the participants’ fingers to measure changes in peripheral arterial tone and oxygen saturation, along with a built-in actigraphy to measure sleep time. The severity of OSA is assessed by PAT Apnea Hypopnea Index (AHI) which is automatically generated by the software, using changes in the peripheral arterial tonometry. OSA is considered present if AHI ≥ 5. Because this device relies on changes in peripheral arterial tone, use of certain medications was not allowed as described in the exclusion criteria.

Participants wore an Actiwatch 2 wrist activity monitor (Philips Respironics, Bend, Oregon) for approximately 7 days (in general, five week days and two weekend days to reflect habitual sleep patterns). These monitors use highly sensitive omnidirectional accelerometers to count the number of wrist movements in 30-s epochs. The software scores each 30-s epoch as sleep or wake based on a threshold of activity counts that is estimated using activity within the epoch being scored as well as the epochs 2 min before and after that epoch. This was based on the automatic algorithm of the manufacturer’s software (ten immobile minutes with a medium activity count threshold of 40). Bedtime and wake time are set by the researcher using the event markers, a sleep log data as well as an in-person review of sleep timing with the participants upon return of the watch. Sleep duration was the amount of actual sleep obtained at night. Sleep efficiency (a measure of sleep quality) was the percentage of time in bed spent sleeping. These two variables were calculated using Actiware 6.0 software, supplied by the manufacturer. Midsleep time was the midpoint between sleep onset and sleep end time. Standard deviation of midsleep time, a marker of sleep variability, was calculated^62^. For each participant, the mean across all available nights was used. For 94.4% of the participants, ≥6 days of recordings were available, while the remaining had between 3–5 days of recordings.

### Dietary assessments

Participants recorded their food intake and timing for 7 days using food diaries. Mealtime was reported as breakfast, lunch, and dinner. Calorie consumption was calculated using a Thai food database (INMUCAL-Nutrients V.3, Institute of Nutrition, Mahidol University, Thailand). Percentages of caloric distribution for each meal and daily caloric intake from fat, carbohydrate and protein were calculated.

### hs-CRP measurements

After an overnight fast, blood samples were obtained. Serum hs-CRP was determined by particle enhanced immunoturbidimetric assay on a Cobas c501 analyzer (Roche Diagnostics, Mannheim, Germany). The intra-assay coefficient of variations were 3.9% and 0.7% at mean concentrations of 0.7 mg/L and 12.2 mg/L, respectively.

### Statistical analyses

Data are presented as mean (standard deviation, SD), median (interquartile range, IQR) or frequency (%) for continuous and categorical data as appropriate. Simple linear regression analyses tested associations between demographics, sleep characteristics, CSM score and hs-CRP. AHI and hs-CRP were natural log (ln) transformed as they were not normally distributed. Predictors of ln hs-CRP were determined using multivariate analysis with backward elimination method. Significant variables associated with hs-CRP from univariate analyses (p < 0.1) were included in the model. Only significant variables (p < 0.05) from multivariate analysis were kept in the final model. The assumption of normality was assessed by the Shapiro-Wilk statistic that had the P-value of 0.865. This revealed that the residuals were normally distributed. A variance inflation factor (VIF) was applied to assess multicollinearity. VIFs of all variables in the final model were close to 1, suggesting that there was no evidence of collinearity. Analyses were performed using STATA 15.0 software. The datasets generated during and/or analyzed during the current study are available from the corresponding author on reasonable request.
